# Studies on the Interaction between Model Proteins and Fluorinated Ionic Liquids

**DOI:** 10.3390/pharmaceutics15010157

**Published:** 2023-01-03

**Authors:** Márcia M. S. Alves, Manuel N. Melo, Haydyn D. T. Mertens, Ana B. Pereiro, Margarida Archer

**Affiliations:** 1 Instituto de Tecnologia Química e Biológica António Xavier, Universidade Nova de Lisboa (ITQB NOVA), 2780-157 Oeiras, Portugal; 2 European Molecular Biology Laboratory (EMBL), Hamburg Unit c/o Deutches Elektronen Synchrotron (DESY), 22607 Hamburg, Germany; 3 Department of Chemistry, NOVA School of Science and Technology, NOVA University Lisbon, 2829-516 Caparica, Portugal

**Keywords:** fluorinated ionic liquids, surface-active ionic liquids, encapsulation, protein

## Abstract

Proteins are inherently unstable, which limits their use as therapeutic agents. However, the use of biocompatible cosolvents or surfactants can help to circumvent this problem through the stabilization of intramolecular and solvent-mediated interactions. Ionic liquids (ILs) have been known to act as cosolvents or surface-active compounds. In the presence of proteins, ILs can have a beneficial effect on their refolding, shelf life, stability, and enzymatic activities. In the work described herein, we used small-angle X-ray scattering (SAXS) to monitor the aggregation of different concentrations of ILs with protein models, lysozyme (Lys) and bovine serum albumin (BSA), and fluorescence microscopy to assess micelle formation of fluorinated ILs (FILs) with Lys. Furthermore, coarse-grained molecular dynamics (CG-MD) simulations provided a better understanding of Lys–FIL interactions. The results showed that the proteins maintain their globular structures in the presence of FILs, with signs of partial unfolding for Lys and compaction for BSA with increased flexibility at higher FIL concentrations. Lys was encapsulated by FIL, thus reinforcing the potential of ILs to be used in the formulation of protein-based pharmaceuticals.

## 1. Introduction

Proteins are highly complex biomolecules that are present in several vital processes. In order to remain active, proteins must maintain their secondary structural elements, which are shaped through a delicate balance between hydrogen bonds, disulfide bridges, and hydrophobic and ionic interactions [1,2]. As a result of these interactions, native globular proteins are densely packed, which discourages non-specific aggregation [3]. Concomitantly, when the protein structure is destabilized, the exposure of the buried hydrophobic domains may lead to non-specific interactions. Although the interaction between proteins is essential for life, an abnormal increase in protein–protein interactions can lead to the unwanted formation of protein aggregates, which play major roles in diseases such as Alzheimer’s, type 2 diabetes, and spongiform encephalopathies [4]. This inherent structural and chemical instability, associated with short half-lives when subjected to physical and chemical stress, limits the use of proteins as therapeutic agents [5]. However, since proteins are stabilized by the equilibrium between intramolecular interactions and interactions with the solvent environment, the employment of biocompatible cosolvents can provide an alternative strategy to preserve their stability [5]. 

Ionic liquids (ILs) are organic salts comprising ions that are liquid at room temperature [2]. ILs display low vapor pressures, low flammability, are thermodynamically stable, and are generally recognized as safe starting materials [1,2]. These properties make ILs very desirable solvents in biocatalysis, extraction, and electrochemistry, as they are able to solvate a broad range of organic substrates [6]. In the presence of proteins, ILs are known to act as cosolvents, with the ability to assist in refolding, increase shelf life, enhance thermal stability, and improve enzymatic reaction rates [5]. The potential for ILs to stabilize and solubilize proteins demonstrates great promise in the formulation of protein-based biopharmaceuticals, since their use is still not widespread due to the aforementioned protein instability [2]. Surface-active ionic liquids (SAILs) in particular display an intrinsic amphiphilic nature due to the presence of long alkyl chains, and their characteristic aggregation behavior enhances the permeability of drugs across biomembranes, acting as better drug carriers than conventional surfactants [2]. The incorporation of SAILs in aqueous solutions facilitates the aggregation and micellization of the ionic liquids, and are excellent additives for proteins, enhancing both structural and chemical stability. However, structure modifications of the proteins, such as BSA, can also lead to denaturation, depending both on the type of side chain and ionic liquid concentration. In BSA, diverse aggregation behavior was observed, depending on the functionalization of these surfactants, which can produce different hydrogen bond capabilities of the SAILs [7]. Fluorinated ILs (FILs) are a specific SAILs family structurally composed of anions or cations with fluorinated alkyl chains equal to or longer than four carbons. These compounds can enhance the specific SAILs properties due to their three different nanosegregation domains: one polar and two different apolar (hydrogenated and fluorinated) [8]. Therefore, FILs can increase the solubilization power, the surfactant power, tuneability, and high thermal stability relative to conventional ILs and perfluoroalkyl compounds (used also as surface-active compounds).

In previous works, we have studied the behavior of model proteins lysozyme (Lys) and bovine serum albumin (BSA) in the presence of biocompatible, non-toxic SAILs [9,10]. We showed that Lys was encapsulated by FILs at concentrations above their critical aggregation concentration (CAC), with no significant impact on the protein thermal stability and activity [9]. In contrast, the stability of BSA improved upon encapsulation by FILs, as shown by the increase in melting temperature (*T*_m_)—verified by multiple thermostability assays—probably due to a more compact folding state, since the protein α-helical content also increased in the presence of FIL [10].

In the work herein presented, small-angle X-ray scattering (SAXS) experiments were conducted for both Lys and BSA to describe the aggregates in the aqueous solution. Coarse-grained molecular dynamics (CG-MD) simulations were performed to better comprehend Lys–FIL interactions, and fluorescence microscopy assays were conducted to provide visual insights into the protein encapsulation by FILs. This fundamental study is essential to understand all interactions present in these complex biological systems, and is the first step to the application of FILs in drug delivery systems (DDSs) for the delivery and stabilization of valuable therapeutic proteins.

## 2. Materials and Methods

### 2.1. Materials

Lyophilized lysozyme from chicken egg white (L6876), lyophilized bovine serum albumin (A7030), potassium chloride, KCl (purity 99.0%, P9333), and potassium phosphate monobasic (purity 99.0%, P0662) were purchased from Sigma-Aldrich; potassium phosphate dibasic (purity 99.0%, P749.3) and HEPES (4-(2-hydroxyethyl)-1-piperazineethanesulfonic acid) buffer (purity 99.5%, 9105) were purchased from Roth.

Cholinium ((2-hydroxyethyl)trimethylammonium) dihydrogen phosphate, [N_1112(OH)_][H_2_PO_4_] (>98% mass fraction purity), cholinium perfluorobutanesulfonate, [N_1112(OH)_][C_4_F_9_SO_3_] (>97% mass fraction purity), and 1-ethyl-3-methylimidazolium perfluorobutanesulfonate, [C_2_C_1_Im][C_4_F_9_SO_3_] (>97% mass fraction purity) were supplied by IoLiTec GmbH. To reduce the volatile chemicals and water contents, all ILs were dried under vacuum (3·10^−2^ Torr) with vigorous stirring at about 323 K for at least 2 days immediately prior to their use. No further purification was carried out and the purity of all ILs was checked by ^1^H and ^19^F NMR. The chemical structures of the ionic liquids used in this work are presented in Table 1. 

Fluorescent reagents Nile Red, NHS-Rhodamine, and NHS-Fluorescein were purchased from Thermo Fisher Scientific, Waltham, MA, USA.

### 2.2. Protein Purification 

Size exclusion chromatography was undertaken in order to isolate the BSA monomer and ensure its monodispersity in the following studies. Briefly, lyophilized BSA was reconstituted in 50 mM HEPES pH 7.5, 50 mM KCl, and loaded onto a HiLoad 16/600 Superdex 200 column (GE Healthcare, Chicago, IL, USA) which was pre-equilibrated in the same buffer. Fractions of 1.5 mL were collected, and those corresponding to the BSA monomer were pooled and flash frozen in liquid nitrogen. Aliquots were thawed, centrifuged (9184× *g*, 10 min, 277.15 K), and used for further experiments. All assays were performed using 50 mM HEPES pH 7.5, 50 mM KCl buffer, unless otherwise stated.

### 2.3. Small-Angle X-ray Scattering

SAXS data were collected at beamline P12 operated by EMBL Hamburg at the PETRA III storage ring (DESY, Hamburg, Germany) [11]. Measurements were performed under constant flow in batch mode. Protein concentrations were 3–4 mg·mL^−1^ for lysozyme (50 mM Tris HCl pH 7.5) and 5 mg·mL^−1^ for BSA (50 mM HEPES pH 7.5). Ionic liquid stock solutions were prepared at 50% *v*/*v* in MilliQ water, and were then added in a concentration range from 0 to 1.8% *v*/*v* to the respective buffers. IL blanks were also measured. Images were recorded using a Pilatus-6M detector at a sample to detector distance of 3.0 m and λ = 0.12 nm, covering the range of momentum transfer 0.01 < s < 7 nm^−1^ (s = 4πsinθ/λ, where 2θ is the scattering angle). Data were processed and analyzed with the ATSAS program suite, version 3.0.3.1 (Hamburg, Germany) [12], using PRIMUS [13] for further subtraction and averaging as required, and for radius of gyration (*R*_g_) and other SAXS invariant estimations. The program OLIGOMER was used for equilibrium analysis of components in solution for BSA, with computed scattering intensities of components (PDB ID: 4F5S) calculated in FFMAKER [13].

### 2.4. Coarse-Grained Molecular Dynamics

The Martini 3 CG model [14] was employed to simulate lysozyme in the presence and absence of FIL and for single proteins or in pairs. The GROMACS v2020 software [15] was used for simulation. CG protein structures and molecular topologies were obtained using the *martinize2* tool [16] and set up in boxes at a final protein/water ratio corresponding to approximately 3 mM for the single-protein runs or 1.5 mM for the two-protein runs; 130 mM FIL was used.

Cholinium parameters were obtained from existing Martini 3 models. Perfluorobutanesulfonate was parameterized following the Martini 3 building block approach, where a sulfate particle (or bead) was connected to two perfluoro beads (each covering two carbons) of type X1e—an addition in Martini 3 that better represents haloalkanes. Beads were constrained at a separation corresponding to their center-of-mass distances when mapped on the fully extended atomic structure of perfluorobutanesulfonate (sulfate–perfluoro at 2.45 Å and perfluoro–perfluoro at 2.8 Å). Analogously to Martini 3 alkanes, the three beads were weakly restrained to 180° by a 50 kJ/mol cosine harmonic potential.

Systems were solvated first with FIL (when used) in a randomly dispersed fashion around the protein(s), and then with copies of equilibrated Martini water boxes. Subsequently, 150 mM NaCl ionic strength was added at this stage, with excess chloride ions to neutralize the protein’s charge. Simulations employed a standard Martini timestep of 20 fs, with nonbonded interactions cut off at 1.1 nm. Reaction field electrostatics with a dielectric constant of 15 were used. Equilibration to 1 bar and 300 K was carried out over 10 ns using the Berendsen barostat [17], for its robustness, and the v-rescale thermostat [18]. Production runs employed the Parrinello–Rahman barostat [19]. Each system was run in triplicate from the equilibration step (when random velocities were assigned to particles) with individual production runs of at least 39 µs for the single-protein systems without FIL, 59 µs with FIL, and 83 µs for the two-protein systems with or without FIL (the total simulation time, over all replicas, was 0.81 ms).

Trajectory analysis was performed using the VMD v1.9.3 visualization software [20] as well as the NumPy [21] and MDAnalysis [22] Python packages. Structure clustering was performed using the algorithm provided by Daura et al. [23]. To correctly cluster the cases of asymmetric dimers, trajectories were analyzed duplicated, with protein identities switched in the duplicated segment.

### 2.5. Preparation of Nile-Red-Loaded Micelles

Nile Red (NR) is an environment-sensitive stain that becomes intensely fluorescent in lipid-rich or hydrophobic environments. NR has been used as a hydrophobic probe to study the local polarity of heterogeneous systems such as micelles, or combined with proteins bearing hydrophobic domains, as in the case of albumins [24,25].

NR encapsulation into FIL micelles was performed by diluting 1 mM NR stock solution in PBS to a final concentration of 10 µM in the presence of different FIL concentrations. These NR-FIL solutions were allowed to equilibrate overnight at RT. Spectra were then measured from 400 to 700 nm using a NanoDrop One with subtraction of the blank buffer to follow NR dye absorbance, and data were extrapolated for 1 cm path length.

### 2.6. Protein Labeling

Protein labeling was performed using N-hydroxysuccinimide (NHS)-ester fluorescent reagents, namely, NHS-Rhodamine and NHS-Fluorescein, which react with primary amines to form stable amide bonds. Reactions were prepared according to the manufacturer’s instructions. Briefly, the dye was dissolved in DMSO and added to lysozyme in phosphate buffer at a 5:1 molar ratio. The solution was then incubated for 1 h at RT, and excess dye was removed using fluorescent dye removal columns (Thermo Fisher Scientific). After labeling, the protein was stored at 277 K prior to use, and protected from light. Dye and protein absorbance were quantified using a NanoDrop One spectrophotometer.

### 2.7. Fluorescence Microscopy

Samples for fluorescence microscopy were applied to microscopy slides coated with 1.7% agarose and observed under a Leica DM 6000B microscope equipped with a phase contrast Uplan F1 100× objective and a CCD Andor Ixon camera (Andor Technologies, Belfast, UK). Images were acquired and analyzed with bright field, T × 2, and FITC filters, according to fluorophore properties (see Table 2), using the Metamorph software suite (Molecular Devices, San Jose, CA, USA).

## 3. Results and Discussion

### 3.1. SAXS

**Figure 1:**
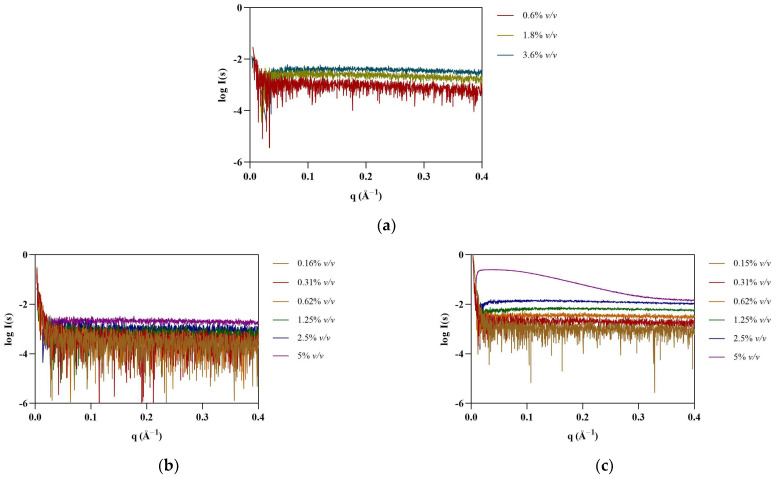
SAXS experimental scattering curves for IL blanks: (**a**) [N_1112(OH)_][H_2_PO_4_], (**b**) [N_1112(OH)_][C_4_F_9_SO_3_], and (**c**) [C_2_C_1_Im][C_4_F_9_SO_3_].

SAXS provides information on the shape and size of biomolecules in solution, making it suitable to study dynamic systems such as protein–IL complexes. SAXS data were first collected for [N_1112(OH)_][H_2_PO_4_], the non-surfactant IL, at 0.6, 1.2, and 1.8% *v*/*v* (see Figure 1). As expected, no assemblies were detected for this IL. Surfactant FILs were used from 0.16 to 5% *v*/*v* in order to evaluate structure formation in this concentration range. Scattering profile for [N_1112(OH)_][C_4_F_9_SO_3_] remained unchanged up to 5% *v*/*v*, whereas [C_2_C_1_Im][C_4_F_9_SO_3_] contributed strongly to the SAXS signal at 5% *v*/*v* (Figure 1). This observation was unexpected, as both FILs have been described to self-assemble into organized nanostructures above their critical aggregation concentration (*CAC*) [8]. Due to the lack of contrast at low concentrations close to the *CMC*, especially for systems that form less compact micelles, characterization of SAILs by SAXS is often reported at much higher concentrations [7]. Since the SAXS signal for [C_2_C_1_Im][C_4_F_9_SO_3_] only occurs at high concentrations (8 × *CAC*), which are well above the concentrations used in our studies, its interference with protein measurements in the presence of ILs (0.3–1.8% *v*/*v*) is considered insignificant. Nevertheless, all IL blank buffers were subtracted from the raw data of the corresponding samples.

**Figure 2:**
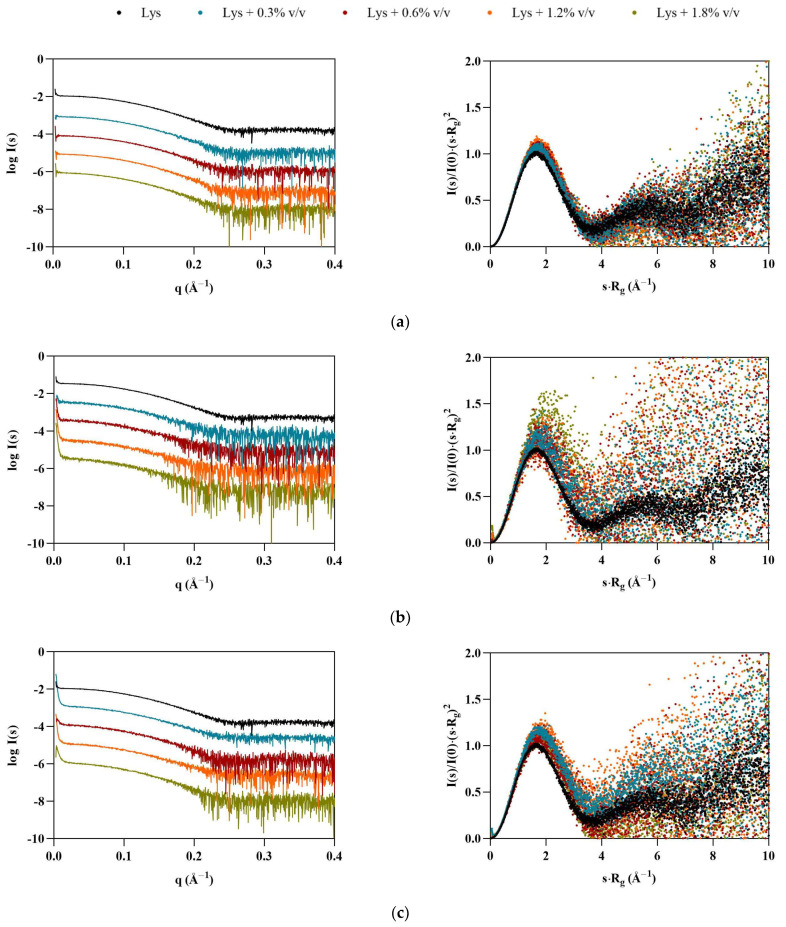
SAXS scaled experimental scattering curves (left) and dimensionless Kratky plots (right) for Lys in the presence of (**a**) [N_1112(OH)_][H_2_PO_4_], (**b**) [N_1112(OH)_][C_4_F_9_SO_3_], and (**c**) [C_2_C_1_Im][C_4_F_9_SO_3_].

Once the profiles of ILs in solution had been established, SAXS data were collected for the model proteins, starting with lysozyme. The shapes of the dimensionless Kratky plots (Figure 2, right) indicate that Lys maintains its globular folded structure upon IL addition. However, the peak shift observable in the presence of the ILs—which is even more apparent in the case of the FILs—suggests that some flexibility may have been introduced to the protein, which is also supported by the increase in noise in the traces for both [N_1112(OH)_][C_4_F_9_SO_3_] and [C_2_C_1_Im][C_4_F_9_SO_3_]. All ILs led to an increase in average Lys particle size (see Table 3), which was overall more significant for the FILs. At 1.8% *v*/*v* [N_1112(OH)_][C_4_F_9_SO_3_], the radius of gyration (*R*_g_) increased by 22%, and maximum particle dimensions (*D_max_*) by 29%, while both Porod volume (*V*_P_) and molecular mass (MM) had a maximum increase of 22% in the presence of 1.8% *v*/*v* [C_2_C_1_Im][C_4_F_9_SO_3_]. The apparent increase in the size of protein is indicative of partial unfolding in IL solutions. Other studies corroborate lysozyme conformational changes and unfolding induced by ILs, which depend on the IL type and concentration [26,27,28].

**Figure 3:**
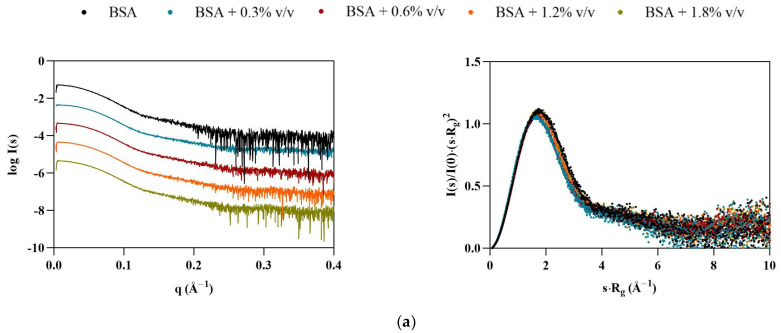

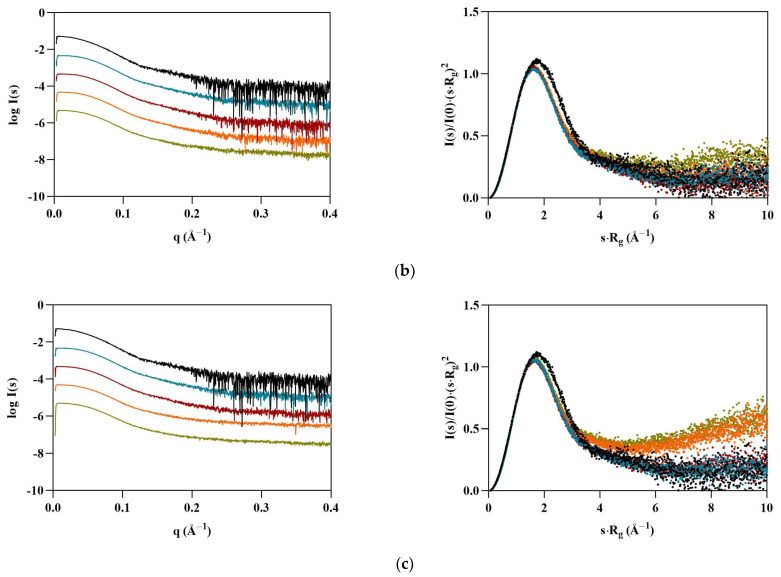
Scaled experimental scattering curves (left) and dimensionless Kratky plots (right) for BSA in the presence of (**a**) [N_1112(OH)_][H_2_PO_4_], (**b**) [N_1112(OH)_][C_4_F_9_SO_3_], and (**c**) [C_2_C_1_Im][C_4_F_9_SO_3_].

Interestingly, BSA displayed the opposite behavior of Lys, with particles appearing to become smaller in the presence of the ILs (Table 4). At 1.8% *v*/*v* [C_2_C_1_Im][C_4_F_9_SO_3_], all calculated structural parameters reached their minimum, with *R*_g_, *V*_P_, *D_max_*, and MM decreasing by 11, 20, 25, and 20%, respectively. The scattering curves for BSA (Figure 3, left) indicate that some interparticle repulsions (downturn shape at low angles) are always present even in the absence of ILs, but become more pronounced with [C_2_C_1_Im][C_4_F_9_SO_3_]. These observations suggest that the solution is becoming partially ordered due to repulsive structure factors. The dimensionless Kratky plots (Figure 3, right) remain characteristic of a globular folded protein, but shift slightly to higher angles, suggesting compaction of the BSA structure, unlike Lys. These results are in agreement with our previous studies [9]. However, diverse aggregation behavior, destabilization, and unfolding of BSA with SAILS molecules has been reported [7,29].

**Figure 4:**
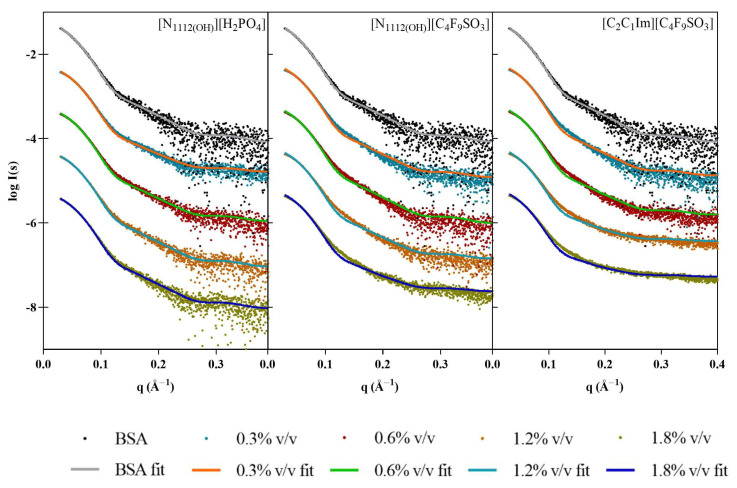
Scaled experimental scattering curves superimposed with fits obtained from OLIGOMER for BSA in the presence of [N_1112(OH)_][H_2_PO_4_], [N_1112(OH)_][C_4_F_9_SO_3_], and [C_2_C_1_Im][C_4_F_9_SO_3_].

Although the BSA samples used in these assays were pooled monomeric fractions isolated after SEC, there were still concerns that some spontaneous dimerization could be biasing the results. In order to account for this possibility, the OLIGOMER software package was employed to analyze the oligomeric equilibrium in the different samples using the crystallographic protein structures (PDB code: 4F5S). Overall, the data are always optimally described as 100% monomer, with no dimers present (Figure 4). In the presence of [N_1112(OH)_][H_2_PO_4_], the small differences between the scattering and the fit may be accounted for by the slight conformational rearrangements and/or changes in flexibility. For the FILs, the visible smearing out of the characteristic BSA minima at 0.12–0.13 Å^−1^ supports possible structure compaction and increased flexibility/conformational polydispersity.

### 3.2. Molecular Dynamics

**Figure 5:**
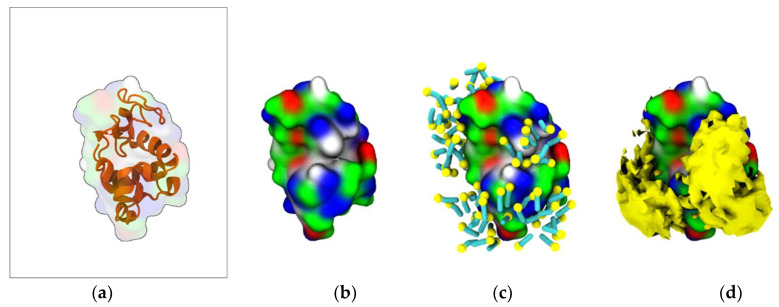
MD simulations of interaction between Lys and [N_1112(OH)_][C_4_F_9_SO_3_]: (**a**) atomistic structure of Lys (PDB ID:1DPX); (**b**) CG structure of Lys; (**c**) [C_4_F_9_SO_3_]^−^ micelles interacting with Lys surface (C_4_F_9_—turquoise, SO_3_—yellow); (**d**) micelle occupancy. Protein surface residues are represented as polar (green), apolar (white), cationic (blue), or anionic (red).

To characterize the interaction between [N_1112(OH)_][C_4_F_9_SO_3_] and Lys, coarse-grained molecular dynamics (CG-MD) simulations were performed. Initially, the simulations consisted of only one Lys molecule in the presence of FIL. As can be seen in Figure 5c, the amphipathic [C_4_F_9_SO_3_]^−^ anions self-assembled into micelles at the surface of the protein. These micelles interacted with both cationic and apolar residues, and were able to occupy the substrate binding cleft (Figure 5d).

**Figure 6:**
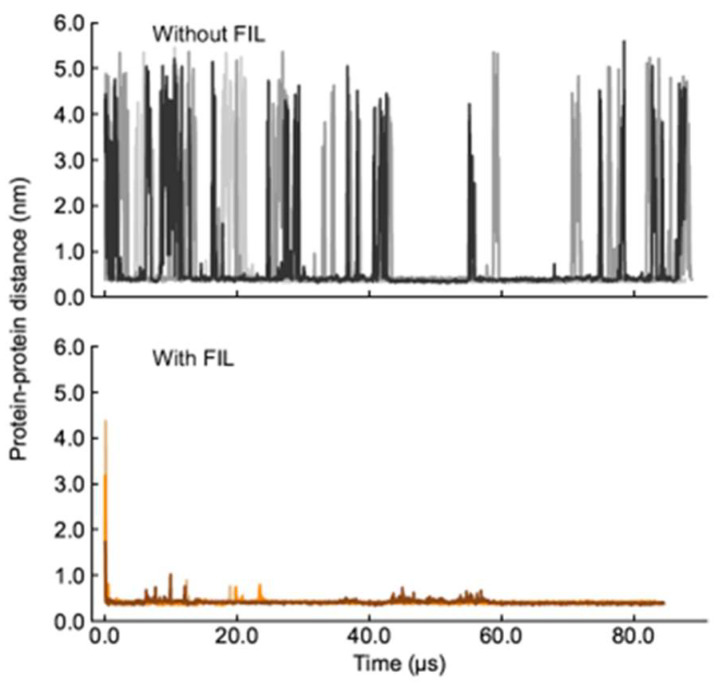
Lys–Lys contacts over time in the absence (**top**) and presence (**bottom**) of [N_1112(OH)_][C_4_F_9_SO_3_].

A second set of simulations was then carried out with two Lys molecules. In the absence of FIL, the two Lys molecules interacted frequently, but always dissociated within the µs timescale (simulations were run too close to 100 µs due to these unbinding timescales). Such behavior was in stark contrast to that when FIL was included. In the presence of [N_1112(OH)_][C_4_F_9_SO_3_], the proteins came together and remained in contact for the entire length of the simulation, only separating to small extents and for brief periods to rearrange (Figure 6).

**Figure 7:**
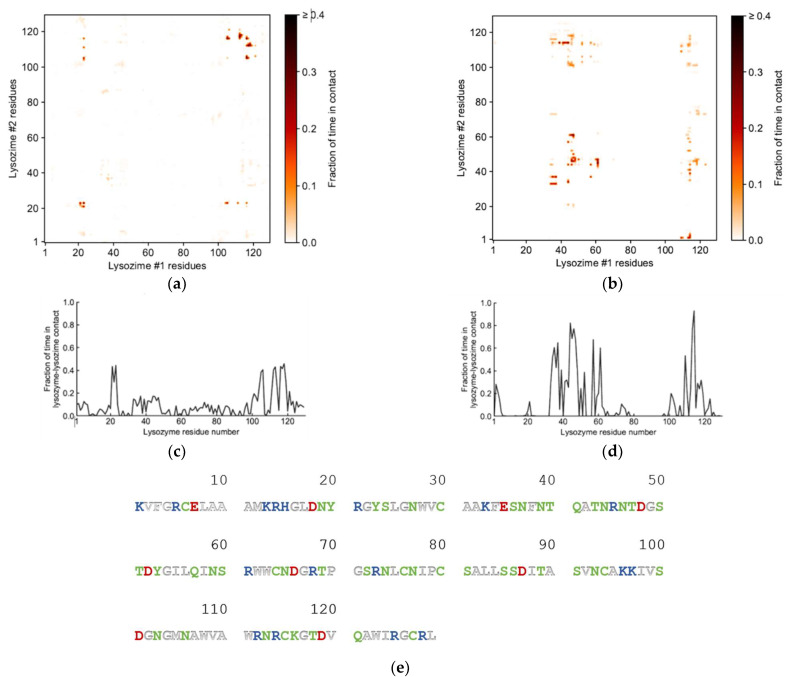
Lys–Lys interactions mediated by [N_1112(OH)_][C_4_F_9_SO_3_]: heat maps of interaction between residues in the absence (**a**) and presence (**b**) of FIL, and corresponding duration of contacts (**c**,**d**); (**e**) sequence for Lys (PDB ID: 1DPX). Residues are represented as polar (green), apolar (white), cationic (blue), or anionic (red).

**Figure 8:**
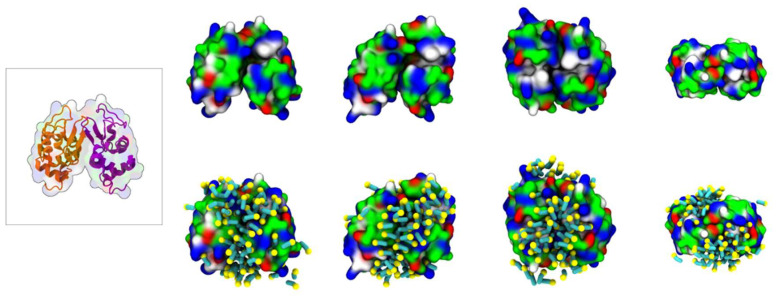
MD simulations of interaction between two Lys molecules and [N_1112(OH)_][C_4_F_9_SO_3_]: atomistic structure of Lys–Lys (PDB ID:1DPX) (**left**); CG structures of Lys–Lys (**top**) and respective [C_4_F_9_SO_3_]^−^ micelle interactions (**bottom**). Protein surface residues are represented as polar (green), apolar (white), cationic (blue), or anionic (red).

**Figure 9:**
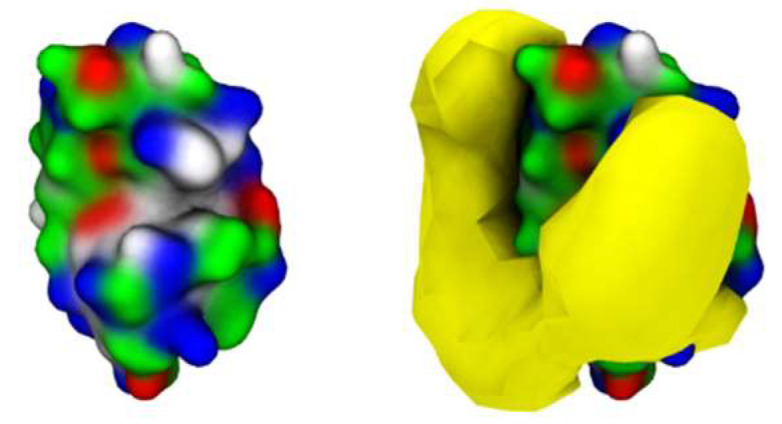
[C_4_F_9_SO_3_]^−^ micelle occupancy in Lys–Lys interface (only one Lys molecule represented for the sake of clarity).

**Figure 10:**
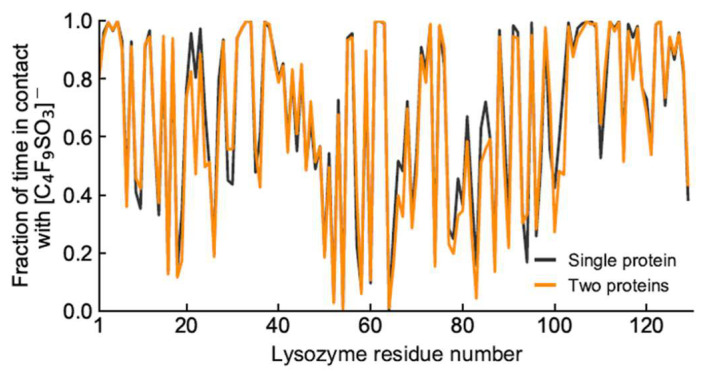
Duration of contacts between Lys residues and [C_4_F_9_SO_3_]^−^.

Further analysis into the contact between both proteins reveals that the residues involved in the interactions are modulated by the presence of FIL (Figure 7). In the absence of FIL, the residues engaged in longer Lys–Lys interactions are mainly 21–24 and 100–120. However, when [N_1112(OH)_][C_4_F_9_SO_3_] was present, the interactions with residues 100–120 (which are mostly apolar) became longer-lived, and residues 35–60 also became involved, even though this is mostly a polar region. Throughout the simulations with FIL, protein structures were similarity clustered, yielding four main Lys–Lys dimerization configurations with different protein orientations (Figure 8) and permanencies. These most common clusters were present for 24, 23, 10, and 7% of the simulation. Independently of how the proteins were oriented towards each other, FIL micelles were always present at the interface, mediating the aggregation. Anion micelle occupancy is represented in Figure 9, which corresponds to the interface of Lys–Lys interactions for at least 40% of the simulation (it is an interface common to more than one of the main clusters, hence the high occupancy). Interestingly, the contact profile between [C_4_F_9_SO_3_]^−^ and Lys residues was not affected by dimerization (Figure 10), reinforcing the idea that the FIL interacts preferentially with specific residues on the protein surface, and is in turn responsible for mediating Lys–Lys aggregation.

### 3.3. Fluorescence Microscopy 

**Figure 11:**
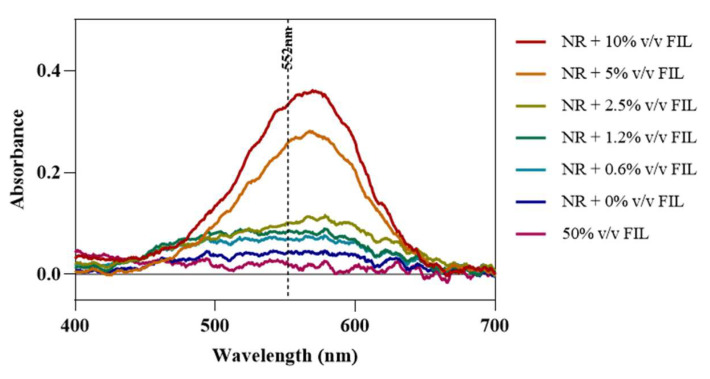
Nile Red absorbance spectra in the presence of increasing concentrations of [C_2_C_1_Im][C_4_F_9_SO_3_]. Vertical line marks NR λ_exc_ (552 nm).

**Figure 12:**
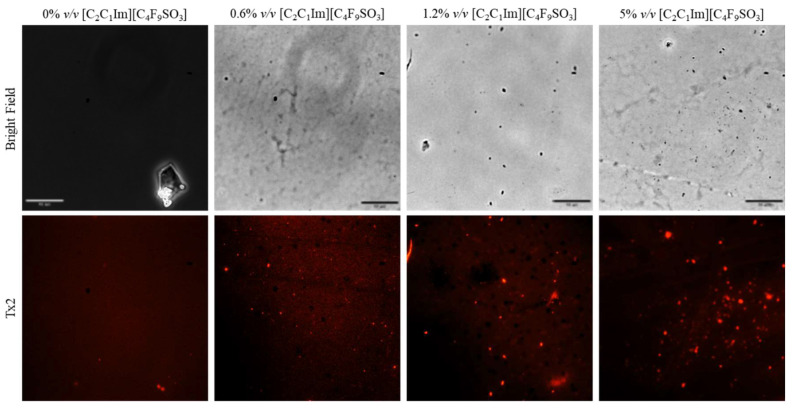
Fluorescence microscopy of Nile Red micelles at 0, 0.6, 1.2, and 5% *v*/*v* [C_2_C_1_Im][C_4_F_9_SO_3_]. Scale bar corresponds to 10 µm.

Fluorescence microscopy was used in an attempt to co-localize Lys within FIL micelles, using different fluorophores. Since tagging the FIL itself would likely lead to significant changes to its properties, we decided to use Nile Red (NR), an environment-sensitive probe, to visualize the micelles. NR only fluoresces in hydrophobic environments, such as the core of micelles. NR was prepared in growing concentrations of [C_2_C_1_Im][C_4_F_9_SO_3_], from 0 to 10% *v*/*v*, and sample absorbance was measured at 552 nm. As can be seen in Figure 11, NR absorbance increased with FIL concentration, which has previously been described for conventional surfactants [25]. When more FIL is available, and consequently more micelles, a higher concentration of NR in the sample is able to absorb light at the characteristic wavelength. Observing these samples under the fluorescence microscope using the T × 2 filter confirmed these results (see Figure 12). Although some fluorescence was detected in the absence of FIL, this may be expected due to the presence of trace amounts of DMSO. In the NR-FIL samples, we can observe a clear correlation between FIL concentration and the number and size of micelles. There are fewer (and smaller) micelles at 0.6% *v*/*v* [C_2_C_1_Im][C_4_F_9_SO_3_], which become larger and more frequent as FIL concentration increases. 

**Figure 13:**
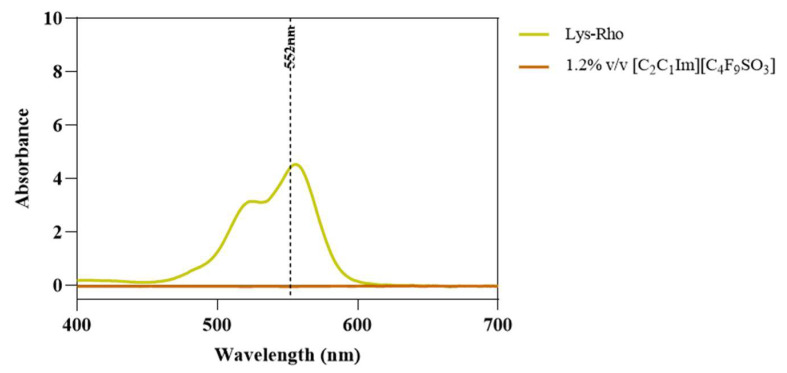
Lys-Rho absorbance spectrum compared to 1.2% *v*/*v* [C_2_C_1_Im][C_4_F_9_SO_3_]. Vertical line marks Rho λ_exc_ (552 nm).

**Figure 14:**
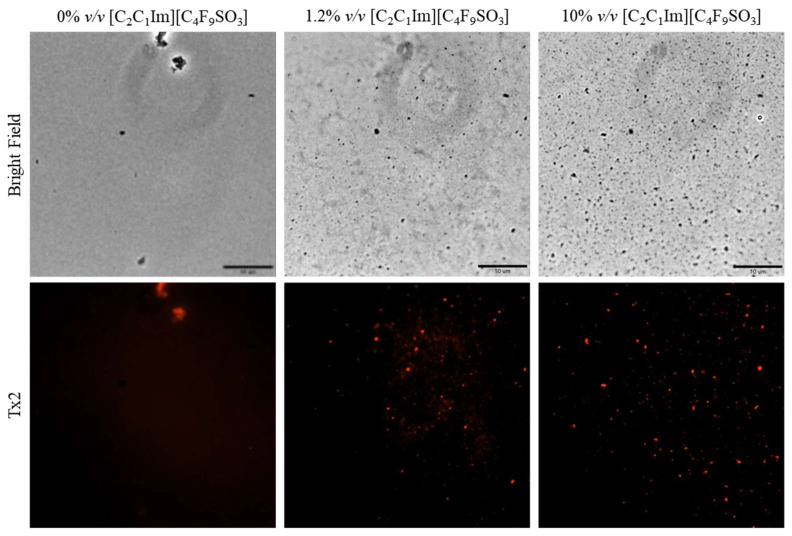
Fluorescence microscopy of Lys-Rho in the presence of 0, 1.2, and 10% *v*/*v* [C_2_C_1_Im][C_4_F_9_SO_3_]. Scale bar corresponds to 10 µm.

**Figure 15:**
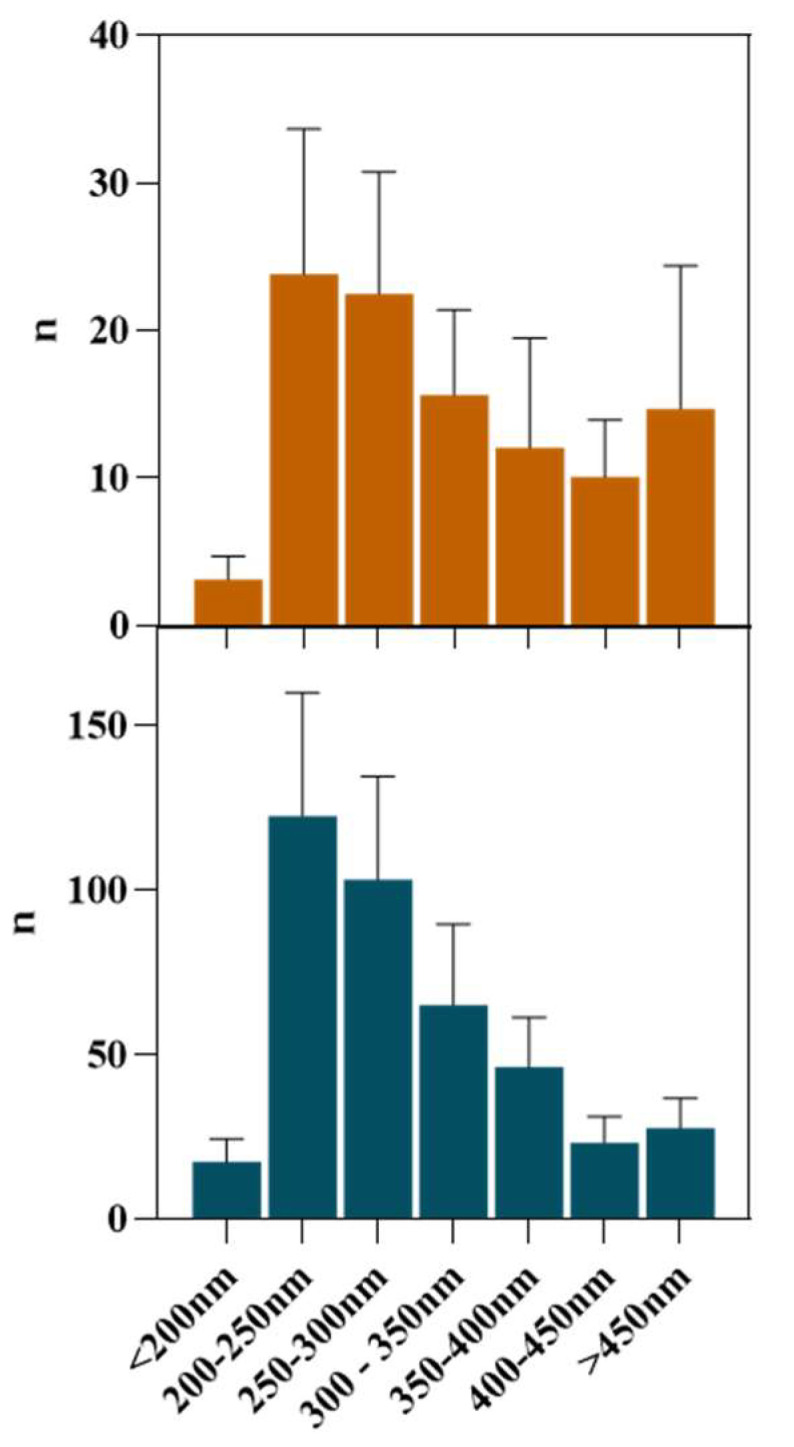
Size distribution of Lys-Rho in the presence of 1.2 and 10% *v*/*v* [C_2_C_1_Im][C_4_F_9_SO_3_].

Once the FIL micelles had been visualized, lysozyme was tagged with NHS-Rhodamine (Rho) to shed some light on its behavior at different [C_2_C_1_Im][C_4_F_9_SO_3_] concentrations. The absorbance of tagged Lys-Rho was measured (see Figure 13) to confirm the efficiency of the labeling reaction and ensure that the FIL did not absorb at the same wavelength. Fluorescence microscopy of these samples (Figure 14) detected non-specific Lys-Rho aggregates in the absence of FIL. At 1.2% *v*/*v* [C_2_C_1_Im][C_4_F_9_SO_3_], small individual particles can be observed, with some fluorescent background. This indicates that Lys-Rho has been encapsulated by the FIL (higher intensity), but not all the protein is contained within micelles, which explains the background signal. When FIL concentration is increased to 10% *v*/*v*, more particles appear, likely encapsulating Lys-Rho, which was in excess at lower FIL concentrations. Counting the fluorescent particles from both samples revealed very similar size distributions, as presented in Figure 15. In total, 14 images were analyzed for 1.2% *v*/*v* FIL, with a total number of particles (n) of 1428, and 9 images for 10% *v*/*v* FIL, where n = 3655. The increase in FIL led to the formation (and consequent detection) of 2.5-fold more particles containing Lys-Rho. The most frequent particle size was in the 200–300 nm range, which accounted for 45 and 56% of total particles in the presence of 1.2 and 10% *v*/*v* [C_2_C_1_Im][C_4_F_9_SO_3_], respectively.

**Figure 16:**
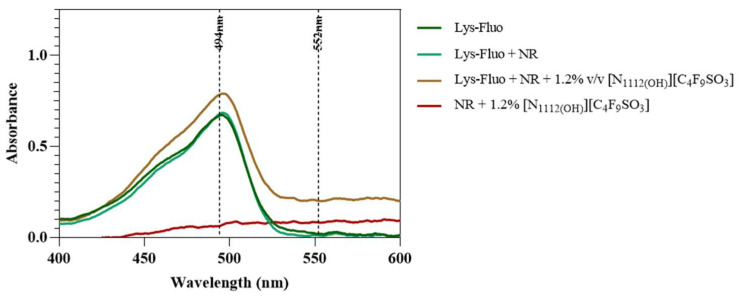
Absorbance spectra of Lys-Fluo + NR samples in the presence of 0 and 1.2% *v*/*v* [N_1112(OH)_][C_4_F_9_SO_3_]. Vertical lines mark Fluo and NR λ_exc_ (494 and 554 nm, respectively).

**Figure 17:**
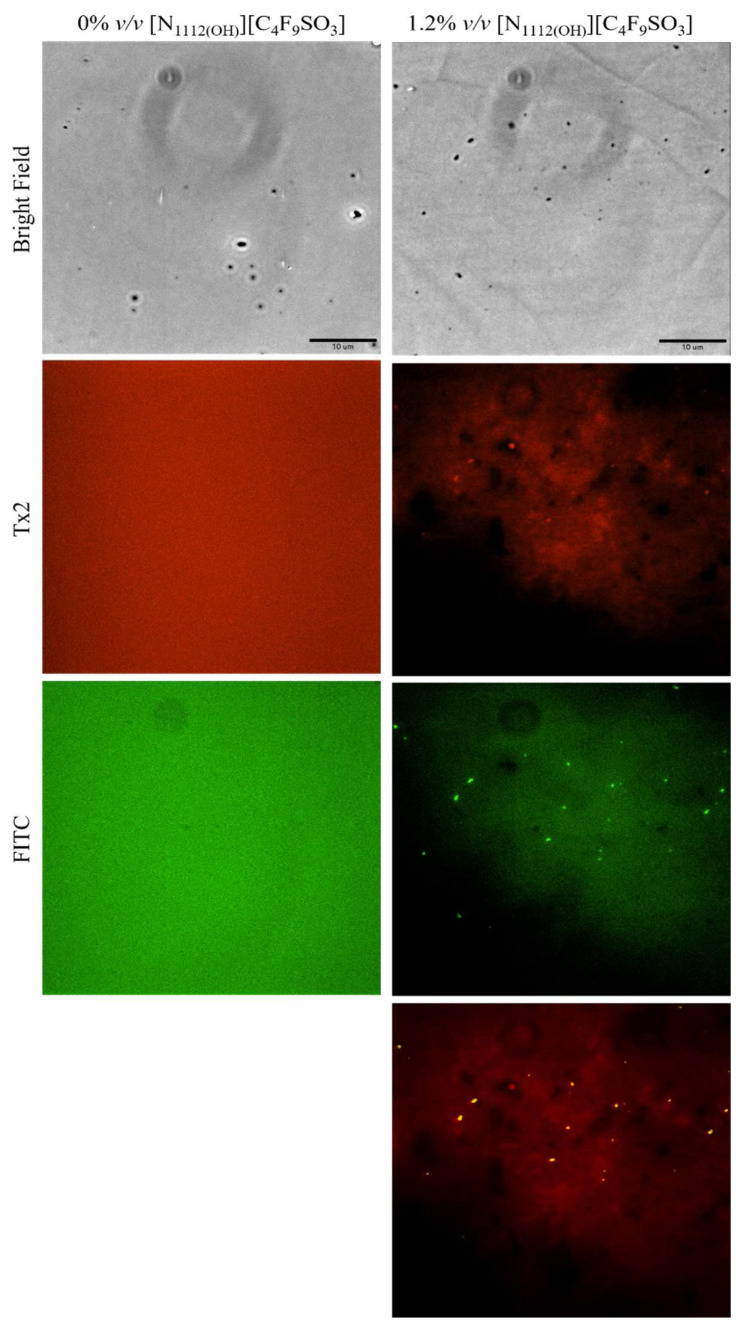
Fluorescence microscopy of Lys-Fluo + NR samples in the presence of 1.2% *v*/*v* [N_1112(OH)_][C_4_F_9_SO_3_]. The last image corresponds to the superposition of the two images above. Scale bar represents 10 µm.

Lastly, lysozyme was tagged with NHS-Fluorescein (Lys-Fluo) instead of NHS-Rhodamine to allow for co-localization with NR, using the [N_1112(OH)_][C_4_F_9_SO_3_] FIL. Absorbance of all samples was measured once again to validate the experimental design (Figure 16). All samples containing Lys-Fluo absorbed light at 494 nm, and only the samples prepared with NR showed absorbance at 552 nm. Fluorescence microscopy images were acquired using two different emission filters, T × 2 and FITC, which correspond to the NR and fluorescein wavelengths, respectively. As expected, FIL micelles were detected using the T × 2 filter, due to NR fluorescence in hydrophobic environments (Figure 17). Lys-Fluo particles were also identified under the FITC filter, with a similar appearance to that previously described for Lys-Rho. Overlaying both fluorescence images allowed us to confirm the co-localization of both the FIL micelles and protein, indicating that Lys is encapsulated by [N_1112(OH)_][C_4_F_9_SO_3_]. Although the overlayed images are not a perfect match in terms of the exact number of particles, this may be due to the presence of NR micelles without protein, or micelles containing Lys-Fluo but not NR, which means that sample concentrations should be optimized.

## 4. Conclusions

The work presented herein further explores the interactions between FILs and model proteins Lys and BSA. IL characterization by SAXS revealed that although both surfactant FILs are known to self-assemble into nanostructures, these were not detected at the studied concentrations. The limitation is caused by a weak contrast at low concentrations (~CMC) of ILs, especially for those forming less compact micelles [7]. Only [C_2_C_1_Im][C_4_F_9_SO_3_] scattered X-rays, but at such high concentrations (8 × *CAC*) that it did not hamper the use of this technique. In the presence of FILs, Lys was shown to maintain its globular folded structure, although some conformational sampling/flexibility may have been introduced. Furthermore, at 1.8% *v*/*v* FIL, all Lys structural parameters had increased, on average, by 24%. In contrast, BSA particles became smaller in the presence of FILs, with an average decrease of 19% for the same concentrations, indicating structure compaction, in agreement with our previous studies [10].

CG-MD simulations were performed for systems containing Lys and [N_1112(OH)_][C_4_F_9_SO_3_] in order to better understand local molecular interactions. These simulations showed [C_4_F_9_SO_3_]^−^ micelles assembling at the surface of the protein, interacting preferentially with cationic and apolar residues, and occupying the binding cleft. However, the experimental data show that Lys maintained (and even increased) its hydrolytic activity in the presence of FILs [8]. Therefore, we can conclude that this interaction does not preclude the enzymatic reaction. When two Lys molecules were present in the simulation box, FIL was shown to mediate their aggregation, being present at the interface of all configurations that were detected. Independently of the number of Lys molecules present, [C_4_F_9_SO_3_]^−^ always interacted with the same residues, mediating protein aggregation. 

Fluorescence microscopy studies led to interesting new insights into FIL micelle formation. NR fluorescence, which is only detected in hydrophobic environments, was dependent on FIL concentrations, and the particles increased not only in number but also in size at higher FIL concentrations. In the presence of Lys-Rho, however, micelle size did not increase with FIL concentration. This suggests that micelle size is defined by the protein rather than the FIL available in the aqueous solution. Tagging Lys with Fluo instead of Rho allowed for co-localization of the protein within FIL-NR micelles, confirming Lys encapsulation, as previously suggested [9].

The experimental data and theoretical studies presented in this work corroborate previously reported effects of FILs on model proteins using other techniques, and provide new insights into the formation of protein–FIL micelles. The essential information obtained in this work opens new paths to the investigation of DDSs based on FILs for therapeutic proteins. These studies are a first and crucial step towards understanding the interactions between FILs and proteins, as well as the discovery of feasible FIL-based DDSs, opening new avenues for the application of FILs in the pharmaceutical industry.

## Figures and Tables

**Table 1:** Chemical structures and acronyms of the ionic liquids (ILs) used in this work.

IL Designation	Chemical Structure	Critical Aggregation Concentration (CAC) in Water
Cholinium dihydrogen phosphate [N_1112(OH)_][H_2_PO_4_]	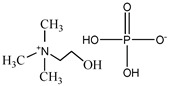	Non-surfactant
Cholinium perfluorobutanesulfonate [N_1112(OH)_][C_4_F_9_SO_3_]	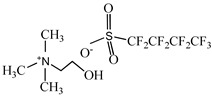	16.07 mM (0.65% *v*/*v*) [8]
1-Ethyl-3-methylimidazolium perfluorobutanesulfonate [C_2_C_1_Im][C_4_F_9_SO_3_]	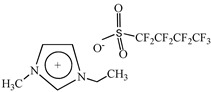	14.55 mM (0.60% *v*/*v*) [8]

**Table 2:** List of fluorophores, respective excitation and emission wavelengths, and molar extinction coefficients.

	Ex/Em (nm)	ε (cm^−1^ M^−1^)
Nile Red (NR)	552/635	38,000
NHS-Rhodamine (Rho)	552/575	80,000
NHS-Fluorescein (Fluo)	494/518	70,000

**Table 3:** SAXS structural parameters of Lys in the absence and presence of ILs.

	*R*_g_ (Å)	*V*_P_ (Å^3^)	*D*_max_ (Å)	MM (kDa)
Lysozyme	14.4 ± 0.00	21,658	42.7	13.54
[N_1112(OH)_][H_2_PO_4_] (% *v*/*v*)				
0.3	14.8 ± 0.00	22,968	44.0	14.35
0.6	14.9 ± 0.00	23,033	46.8	14.40
1.2	15.1 ± 0.00	24,239	47.0	15.15
1.8	15.3 ± 0.01	25,188	48.8	15.74
[N_1112(OH)_][C_4_F_9_SO_3_] (% *v*/*v*)				
0.3	15.4 ± 0.02	22,889	49.1	14.31
0.6	15.4 ± 0.02	22,966	53.0	14.35
1.2	15.7 ± 0.03	24,559	40.8	15.35
1.8	17.6 ± 0.03	23,404	55.0	14.63
[C_2_C_1_Im][C_4_F_9_SO_3_] (% *v*/*v*)				
0.3	15.3 ± 0.01	21,963	50.6	13.73
0.6	15.8 ± 0.01	25,171	51.0	15.73
1.2	15.7 ± 0.01	21,578	50.9	13.49
1.8	15.9 ± 0.01	26,523	51.3	16.58

Radii of gyration (***R*_g_**) were estimated from the Guinier approximation. Excluded particle volumes (***V*_P_**) were estimated from the Porod approximation. Maximum particle dimensions (***D*_max_**) were obtained from the pair distribution function. Molecular mass (**MM**) values were derived from the Porod volume as MM = *V*_P_/1.6.

**Table 4:** SAXS structural parameters of BSA in the absence and presence of ILs.

	*R*_g_ (Å)	*V*_P_ (Å^3^)	*D*_max_ (Å)	MM (kDa)
BSA	29.5 ± 0.01	111,818	101.7	69.89
[N_1112(OH)_][H_2_PO_4_] (% *v*/*v*)				
0.3	27.5 ± 0.02	98,843	82.4	61.78
0.6	27.0 ± 0.00	95,974	79.0	59.99
1.2	27.7 ± 0.00	97,216	85.1	60.76
1.8	28.0 ± 0.00	97,685	91.1	61.05
[N_1112(OH)_][C_4_F_9_SO_3_] (% *v*/*v*)				
0.3	26.4 ± 0.01	95.236	83.2	59.52
0.6	26.3 ± 0.01	93,533	81.4	58.46
1.2	26.5 ± 0.01	91,602	79.9	57.25
1.8	26.1 ± 0.01	90,896	78.5	56.81
[C_2_C_1_Im][C_4_F_9_SO_3_] (% *v*/*v*)				
0.3	26.5 ± 0.01	93,024	79.9	58.14
0.6	26.4 ± 0.01	92,497	79.1	57.81
1.2	26.3 ± 0.01	90,556	78.8	56.60
1.8	26.3 ± 0.01	89,995	76.6	56.25

Radii of gyration (***R*_g_**) were estimated from the Guinier approximation. Excluded particle volumes (***V*_P_**) were estimated from the Porod approximation. Maximum particle dimensions (***D*_max_**) were obtained from the pair distribution function. Molecular mass (**MM**) values were derived from the Porod volume as MM = *V*_P_/1.6.

## Data Availability

Not applicable.
